# Conspiratorial Beliefs About COVID-19 Pandemic - Can They Pose a Mental Health Risk? The Relationship Between Conspiracy Thinking and the Symptoms of Anxiety and Depression Among Adult Poles

**DOI:** 10.3389/fpsyt.2022.870128

**Published:** 2022-06-07

**Authors:** Paweł Dȩbski, Adrianna Boroń, Natalia Kapuśniak, Małgorzata Dȩbska-Janus, Magdalena Piegza, Piotr Gorczyca

**Affiliations:** ^1^Department of Psychiatry, Faculty of Medical Sciences in Zabrze, Medical University of Silesia in Katowice, Tarnowskie Góry, Poland; ^2^Department of Physical Activity and Health Prevention, Institute of Sport Sciences, The Jerzy Kukuczka Academy of Physical Education in Katowice, Katowice, Poland

**Keywords:** COVID-19, conspiracy thinking, conspiratorial beliefs, anxiety, depression

## Abstract

The aim of the study was to describe the relationship between the tendency to believe in false information about the COVID-19 pandemic, tendency to believe in conspiracy theories and the severity of anxiety and depression symptoms among the surveyed Poles. The study was conducted via the Internet in a group of 700 people aged 24.8 ± 6.3 years (mean ± SD). 585 females and 110 males were involved. Scales such as Generic Conspiracist Beliefs Scale (GCBS), Hospital Anxiety and Depression Scale (HADS) and the original questionnaire COVID-19 Conspiratorial Beliefs Scale (COVID-19 CBS) designed to measure the tendency to believe in false information about COVID-19 pandemic were used. A positive correlation was observed between the tendency to believe in false information about the COVID-19 pandemic (COVID-19 CBS) and the tendency to believe in general conspiracy theories (GCBS) (r = 0.768; *p* < 0.001). Moreover, both COVID-19 CBS and GCBS positively correlated with the severity of anxiety and depression symptoms in the study group. For COVID-19 CBS, the correlation coefficients were 0.087 (*p* < 0.021) and.108 (*p* < 0.004) for depressive and anxiety symptoms, respectively, while for GCBS the coefficients were 0.132 (*p* < 0.001) and 0.147 (*p* < 0.001). Regression analysis showed that the increased tendency to believe in false beliefs about the COVID-19 pandemic may be associated with an increase in the severity of anxiety (b = 0.04; *p* = 0.021) and depression (b = 0.06; *p* < 0.001) symptoms. It can be hypothesized that the tendency to believe in false information regarding the COVID-19 pandemic is positively associated with the tendency to general belief in conspiracy theories. False beliefs about the COVID-19 pandemic may, at least to some extent, influence the development of anxiety and depression symptoms.

## Introduction

COVID-19 disease, first diagnosed in China's Hubei province in November 2019, has had a huge impact on people's lives in recent years. The pandemic has brought many changes and restrictions to everyday life that have not been easy for us to get used to. At the same time information about new infections and deaths prevailed in the media has intensified feeling of helplessness and anxiety. People driven by fear of SARS-CoV-2 infection as well as fear of health and life of loved ones, significantly reduced the number of social contacts. Prolonged social isolation and the overall threat of the pandemic have contributed to increased emotional tension and deterioration in the mental health of many people in various parts of the world (1–6).

Research conducted among the population of various countries confirmed the significant impact of COVID-19 pandemic on the mental health of citizens in the context of the severity of anxiety and depressive symptoms. According to the available studies, the prevalence of depressive symptoms in different populations ranged between 14.6% and even 48.3%, while the prevalence of anxiety symptoms oscillated between 6.3% and 50.9% (7). Study conducted in a group of adult Poles revealed a significant intensification of depressive symptoms among 26% respondents and anxiety symptoms among 63% respondents (8). It has also been shown that groups particularly exposed to pandemic distress are women, young people (18–29 years of age), people experiencing mental disorders in the past and people from socially disadvantaged backgrounds (9). Luo et al. noted that the prevalence of anxiety and depressive symptoms is the highest among COVID-19 patients with comorbid conditions, while similar among the other two groups compared: the general population and health care workers (10). A particular group exposed to the negative psychological consequences of the pandemic are young people and children. This group suffered not only from limited contact with peers, but was also exposed to interpersonal violence due to staying at home and escalating parental conflicts during lockdown (11, 12). It is worth mentioning that Heitzman in his work on the mental health impact of the COVID-19 pandemic distinguished the term pandemic acute stress disorder, whose diagnostic criteria largely correspond to the diagnosis of acute stress disorder (13). In addition, it has been estimated from previous research on post-traumatic stress disorder (PTSD) that remote and treatment-requiring mental disorders resulting from exposure to a biological stressor - the SARS-CoV-2 virus - could affect 20% or even a larger proportion of the population.

The COVID-19 pandemic has also become the subject of intense discussion on social media platforms, exposing the danger of the rapid virtual spread of not only reliable and verified information but also so-called “fake news” that may affect the behaviour of the recipients of this content and their compliance with sanitary-epidemiological recommendations (14). Banai et al. point out that COVID-19-related conspiracy beliefs are directly related to non-compliance with health regulations. Furthermore, COVID-19-related conspiracy beliefs are indirectly related with lowering trust in government information (15). Some studies also found a negative relationship between COVID-19 conspiracy beliefs and COVID-19 health-protective behaviours including greater unwillingness to take up future tests and treatment or to be vaccinated (16, 17).

As Rovetta et al. point out, it has become common to adopt generic and stigmatising names for COVID-19 (such as corona, epidemic, influenza wuhan), which in turn can encourage the spread of misinformation. Moreover, generic and stigmatising names were used not only by mass media and Google users, but also by national health agencies and scientists (18). Mass media such as popular social networks have contributed to the spread of false information about the pandemic, increasing fear and anxiety (19). In addition, Internet users sharing false information about the pandemic used other news articles and YouTube videos containing incorrect claims to support their thesis (20).

People who use social media platforms seem to accept conspiratorial narratives more easily compared to people who rarely use this source of information (21). Conspiracy theories regarding the origin of the SARS-CoV-2 virus have become particularly popular. Among the conspiracy theories prevalent were those suggesting that the virus was produced in a laboratory or to reduce the world's population by reducing the number of elderly people. There were also local conspiracy narratives about governments withholding information about the true extent of the pandemic and restricting number of infected people in the spring of 2020 in order, for example, to hold elections. The belief in conspiracy theories related to the course, treatment and origin of the SARS-CoV-2 virus has become a significant health risk, linked to COVID-19 prophylaxis, through which a detailed list of pandemic myths has also been provided by the World Health Organization (WHO) (22).

The social crisis led by the COVID-19 pandemic is associated with an increased tendency to believe in conspiracy theories (23, 24). The feeling of being out of control in the face of a social crisis heightens the need to seek explanation and meaning for an unfamiliar situation, which in turn increases the susceptibility to conspiracy theories, not only those related to the COVID-19 pandemic, but also general ones about how the world works, and is linked to pseudoscientific beliefs (25).

Conspiracy theories have accompanied mankind for many centuries and have concerned various significant events, yet defining this phenomenon can present many difficulties due to the multiplicity and diversity of views regarding its meaning. Aaronovitch et al. defined conspiratorial thinking as unnecessarily assuming the presence of a conspiracy, while another explanation is more likely (26). Another definition refers to an attempt to explain an event or phenomenon by referring to the role of powerful and influential people who try to conceal their involvement, at least until their stated goals are achieved (27). People seem to be more sympathetic to conspiracy narratives when they satisfy important psychological social motives, such as epistemic needs (e.g. the need for understanding and certainty), existential needs (e.g. the need for control and security) and social needs (e.g. the desire to maintain a positive self- or group image) (28). The question remains, however, whether a sense of security based on belief in false claims is not illusory and ultimately displaced by the anxiety associated with not basing one's attitude towards a pandemic on a solid foundation developed by the scientific community. The illusory nature of a security based on a questionable belief system is suggested by previous research indicating that a tendency towards conspiracy thinking is associated with higher levels of anxiety and depressive symptoms (29, 30).

The predicted mental health risks caused by the pandemic have given rise to a study of both protective factors in psychological research, oscillating around mental flexibility (31, 32), and factors that promote mental deterioration through the development of anxiety and depression symptoms. It seems that belief in conspiracy theories belongs to the second of these factors, which is what the present study decided to investigate.

## Aim

The aim of the study was to determine the relationship between the tendency to believe in false information about the COVID-19 pandemic, the general tendency to believe in conspiracy theories and the intensity of anxiety and depression symptoms among the Poles surveyed. We asked whether the general tendency to believe in the most popular conspiracy theories may be a predictor of the tendency to believe in false beliefs about the COVID-19 pandemic and whether the increase in this tendency may result in negative psychopathological phenomena such as increased anxiety and depression symptoms.

## Materials and Methods

The survey was conducted via the Internet between November 2020 and January 2021, addressing a prepared questionnaire to adult Poles (convenience sampling via social media). The questionnaire was completed by 700 people aged 24.8 ± 6.3 years (mean ± SD) who use social media. The study involved 585 females and 110 males, mainly in early and middle adulthood, mostly living in large cities and having a secondary education in most cases. Basic socio-demographic information about the respondents is presented in Table 1.

**Table 1:** Demographic features of the surveyed group of poles (*N* = 700).

**Variable**	**Category**	**Number of respondents**	**Percent of respondents**
Sex	Female	585	83.6%
	Male	110	15.7%
	Other	5	0.7%
Age	18–29 years	618	88.3%
	30–50 years	75	10.7%
	>50 years	7	1.0%
Residence	Village	161	23.0%
	City ≤ 100 thous. of residents	171	24.4%
	City > 100 thous. of residents	368	52.6%
Education	Primary	14	2.0%
	Vocational	4	0.6%
	Secondary	353	50.4%
	Higher	329	47.0%
Marital status	Single	317	45.3%
	In a partnership	310	44.3%
	Married	73	10.4%
Living	Alone	66	9.4%
	With somebody	634	90.6%

Psychological tests and scales with recognised psychometric characteristics and research tradition were used to examine the tendency to believe in conspiracy theories and the severity of anxiety and depression symptoms. A novel, original instrument was used to examine the tendency to believe in false information about the COVID-19 pandemic.

### Generic Conspiracist Beliefs Scale (GCBS)

This test was developed by Brotherton, French, and Pickering (33) to examine the severity of the tendency to believe in conspiracy theories. Polish adaptation studies have estimated that this scale has high internal consistency, which is expressed by an α-Cronbach's coefficient of 0.93 (34). In the sample collected in our study, the α-Cronbach's coefficient for the whole scale was 0.94; 95% CI 0.93–0.94. The reliability of the subscales varies between 0.73 and 0.89. The questionnaire consists of 15 questions, which are answered on a 5-point Likert scale. Testing with this scale allows us to determine the overall intensity of the tendency to believe in conspiracy theories, as well as to estimate five factors: government malfeasance (criminal activity by government organisations), malevolent global conspiracies (secret group exercising control over global events), extraterrestrial cover up (hiding contact with extraterrestrial civilisations), personal well-being (conspiracy theories about individual health and freedom), control of information (unethical control of information flow by various global organisations). The most common tendencies regarding belief in conspiracy theories come down to the factors included in this tool.

### Hospital Anxiety and Depression Scale (HADS)

It is a popular scale in both research and clinical practice developed by Zigmond and Snaith that examines the severity of anxiety and depression symptoms (35). This scale consists of 14 questions, 7 of which concern anxiety symptoms and another 7 depression symptoms. The possible number of points for each question is in the range of 0–3, while the maximum number of points possible to obtain in each subscale is 21. α-Cronbach's coefficient for the subscale of depression symptoms is 0.82, and for anxiety 0.83 (36). In Polish studies, the Cronbach's α coefficient was 0.74 for depression and 0.85 for anxiety (37). In our study, the α-Cronbach's coefficient was 0.76; 95% CI 0.73–0.77 for depression and 0.84; 95% CI 0.82–0.86 for anxiety.

### COVID-19 Conspiratorial Beliefs Scale (COVID-19 CBS)

This is an original questionnaire developed by Debski, Boroń, Kapuśniak, Debska-Janus and Piegza, designed to measure the severity of the tendency to believe in false information about the COVID-19 pandemic. The scale consists of 10 statements related to myths about the COVID-19 pandemic described by the World Health Organization (22) that were popular in social media. Respondents rate how much they agree with the given statements on a 5-point Likert scale (1–5). The obtained points from subsequent items of the questionnaire are added up, the final score is in the range of 5–50 points. The higher the score, the stronger the tendency to believe in false beliefs about the pandemic. The scale is characterised by satisfactory internal consistency - α-Cronbach's coefficient was.87; 95% CI 0.86–0.89. The presented study also confirmed the convergent validity of the scale by relating it to other measures of the tendency to believe in conspiracy theories. Factor analysis, on the other hand, argues for maintaining the two-factor structure of the tool. Therefore, we can use the scale to obtain an overall score or results in two of its subscales - conspiracy beliefs about the harmful effects of humans in the COVID-19 pandemic (α-Cronbach's coefficient was 0.85; 95% CI 0.83–0.87) and false beliefs about prevention and treatment in the conditions of the COVID-19 pandemic (α-Cronbach's coefficient was 0.78; 95% CI.75–0.81). Only the tool's overall score was used in this study. The Polish version of the scale as well as its translation into English can be found in Supplementary Material. Supplementary Material also contain data on the initial study of the psychometric properties of the scale.

### Statistical Analysis

The statistical analysis was performed using the computer programs Excel 2016 and Statistica version 13.3. To assess the normality of the distributions, skewness, and kurtosis were used. Correlation matrix was constructed using Pearson's correlation coefficient. Regression analyses were performed using simple and multiple regression. The U Mann-Whitney test and Kruskal-Wallis test were used to assess the significance of intergroup differences. Cronbach's alpha coefficients for scales used were determined in the study group. The assessment of the significance level was formulated in such a way that: *p* ≤ 0.05 was considered as moderate, *p* ≤ 0.01 as high, and *p* ≤ 0.001 as very high significance.

The initial psychometric properties of the COVID-19 CBS were estimated using Cronbach's alpha coefficient and factor analysis with the use of the Kaiser criterion, the Cattel scree plot and the Varimax method. The discriminant power of the test items was estimated based on the correlation of the items with the overall result, as well as by examining the significance of differences between extreme groups (see Supplementary Material).

Bioethics committee approval was obtained for the study. The study instruction included information on informed consent. The subjects were informed about the anonymity of participation in the study, its voluntariness, and the possibility to freely withdraw from participation at any time. The instructions also included information about the scientific purpose of the study.

## Results

### Descriptive Statistics on the Variables

The basic descriptive characteristics of the variables are presented in Table 2.

**Table 2:** Descriptive statistics of the variables for surveyed group of poles (*N* = 700).

**Variable**	**M**	**SD**	**SK**	**K**
COVID-19 CBS	21.6	8.4	0.56	−0.41
GCBS	37.3	13.7	0.28	−0.53
GCBS - GM	8.0	3.2	0.14	−0.73
GCBS - MGC	7.7	3.5	0.29	−0.99
GCBS - ECU	5.4	3.1	1.14	0.45
GCBS - PW	7.3	3.3	0.38	−0.76
GCBS - CoI	8.9	3.2	−0.10	−0.70
HADS - A	9.6	4.3	0.24	−0.41
HADS - D	5.8	3.7	0.78	0.63

*COVID-19 CBS, COVID-19 Conspiratorial Beliefs Scale-Total score; GCBS, Generic Conspiracist Beliefs Scale, Total score; GM, Government Malfeasance; MGC, Malevolent Global Conspiracies; ECU, Extraterrestrial Cover Up; PW, Personal Well-being; CoI, Control of Information; HADS, Hospital Anxiety and Depression Scale; A, Anxiety; D, Depression; M, mean; SD, standard deviation; SK, Skewness; K, Kurtosis*.

### Associations Connecting Belief in False Information About the COVID-19 Pandemic (COVID-19 CBS), Belief in General Conspiracy Theories (GCBS) and Severity of Anxiety and Depression Symptoms (HADS)

The analysis of the obtained results made it possible to observe the presence of relationships between the studied variables (Table 3). A positive correlation was observed between the tendency to believe in false information about the COVID-19 pandemic (COVID-19 CBS) and the tendency to believe in general conspiracy theories (GCBS). There was also a positive correlation between COVID-19 CBS and all components of GCBS. The associations of COVID-19 CBS with the overall GCBS score, as with its components - belief in unethical control of information by global organisations (GCBS - CoI), belief in conspiracy theories about individual health and freedom (GCBS - PW), belief in control of global events by a secret group (GCBS - MGC), belief in hiding contact with extraterrestrial civilisations (GCBS - ECU), and belief in criminal activity by government organisations (GCBS - GM) proved to have very high significance. The tendency to believe in false beliefs about pandemic (COVID-19 CBS) correlated most strongly with belief in conspiracy theories about individual health and freedom (GCBS - PW).

**Table 3:** Correlations between COVID-19 CBS, GCBS and the severity of anxiety and depression symptoms in a group of Polish respondents (*N* = 700).

**Variable**	**Correlation Coefficient**	**COVID-19 CBS**	**GCBS**	**GCBS-GM**	**GCBS-MGC**	**GCBS-ECU**	**GCBS-PW**	**GCBS-CoI**	**HADS-A**	**HADS-D**
COVID-19 CBS	r	1.000	0.768	0.586	0.676	0.508	0.766	0.700	0.087	0.108
	*p*		<0.001	<0.001	<0.001	<0.001	<0.001	<0.001	0.021	0.004
GCBS	r		1.000	0.835	0.878	0.735	0.896	0.877	0.132	0.147
	*p*			<0.001	<0.001	<0.001	<0.001	<0.001	<0.001	<0.001
GCBS-GM	r			1.000	0.662	0.512	0.646	0.706	0.130	0.147
	*p*				<0.001	<0.001	<0.001	<0.001	<0.001	<0.001
GCBS-MGC	r				1.000	0.524	0.773	0.721	0.093	0.122
	*p*					<0.001	<0.001	<0.001	0.013	0.001
GCBS-ECU	r					1.000	0.589	0.509	0.098	0.113
	*p*						<0.001	<0.001	0.010	0.003
GCBS-PW	r						1.000	0.770	0.112	0.107
	*p*							<0.001	0.003	0.005
GCBS-CoI	r							1.000	0.124	0.133
	*p*								0.001	<0.001
HADS-A	r								1.000	0.631
	*p*									<0.001
HADS-D	r									1.000
	*p*									

*COVID-19 CBS, COVID-19 Conspiratorial Beliefs Scale-Total score; GCBS, Generic Conspiracist Beliefs Scale, Total score; GM, Government Malfeasance; MGC, Malevolent Global Conspiracies; ECU, Extraterrestrial Cover Up; PW, Personal Well-being; CoI, Control of Information; HADS, Hospital Anxiety and Depression Scale; A, Anxiety; D, Depression; r, Pearson's correlation coefficient*.

Correlation analysis also showed that both COVID-19 CBS and GCBS, positively correlated with the severity of anxiety and depression symptoms in the study group. Taking into account the relationship of COVID-19 with anxiety, a moderate significance was obtained, while with depression symptoms, it reached a high significance. In the case of GCBS relationship with the severity of anxiety and depression symptoms, the significance turned out to be very high.

### Belief in General Conspiracy Theories (GCBS) and Belief in False Information About the COVID-19 Pandemic (COVID-19 CBS)

Regression analysis showed the possibility of predicting the severity of belief in false information about the COVID-19 pandemic (COVID-19 CBS) in the light of the components of the general tendency to believe in conspiracy theories, measured by the GCBS scale. The relationship of very high significance was obtained in categories such as: extraterrestrial cover up (GCBS - ECU), personal well-being (GCBS - PW), and control of information (GCBS - CoI). The association with malevolent global conspiracies (GCBS - MGC) turned out to be of moderate significance (Table 4).

**Table 4:** Regression model of belief in false information about the COVID-19 pandemic in the light of the tendency to believe in general conspiracy theories (*N* = 692).

**Predictors**	**Beta**	**b**	**b SE**	***p***
Constant		4.39	0.56	0.003
GCBS, MGC	0.11	0.27	0.09	0.027
GCBS, ECU	0.06	0.17	0.08	<0.001
GCBS, PW	0.49	1.23	0.11	<0.001
GCBS, CoI	0.22	0.58	0.10	<0.001
R^2^ = 0.65; F(4, 687) = 317.42; *p* < 0.001; SEE = 4.92; f2 = 1.86				

*GCBS, Generic Conspiracist Beliefs Scale MGC, Malevolent Global Conspiracies; ECU, Extraterrestrial Cover Up; PW, Personal Well-being; CoI, Control of Information; SE, standard error; R^2^, corrected R-squared; SEE, error of estimation; f^2^, effect size for regression*.

### Belief in False Information About the COVID-19 Pandemic (COVID-19 CBS) and the Severity of Anxiety and Depression Symptoms (HADS)

In a regression analysis capturing the associations of COVID-19 CBS with the severity of anxiety and depression symptoms, it was observed that an increase in the tendency to believe in false information about the COVID-19 pandemic may be associated with an increase in the severity of anxiety (moderate significance) and depression symptoms (very high significance) (Table 5).

**Table 5:** Regression model of anxiety and depression symptoms severity in the light of belief in false information about the COVID-19 pandemic (*N* = 700 and *N* = 689).

**Predictors**	**Beta**	**b**	**b SE**	***p***
HADS-A				
Constant		8.67	0.44	<0.001
COVID-19 CBS	0.09	0.04	0.02	0.021
R^2^ = 0.01; F(1, 698) = 5.32; *p* = 0.021; SEE = 4.25; f^2^ = 0.01				
HADS-D				
Constant		4.33	0.36	<0.001
COVID-19 CBS	0.14	0.06	0.02	<0.001
R^2^ = 0.02; F(1, 687) = 13.54; *p* < 0.001; SEE = 3.42; f^2^ = 0.02				

*COVID-19 CBS, COVID-19 Conspiratorial Beliefs Scale; SE, standard error; R^2^, corrected R-squared; SEE, error of estimation; f^2^, effect size for regression*.

### Intergroup Differences

COVID-19 CBS was differentiated taking the place of residence of the respondents into account. There was a difference between rural residents and residents of a city up to 1,00,000 inhabitants (Me = 24 vs. Me = 20; *p* = 0.008), and rural residents and those living in a city over 100,000 inhabitants (Me = 24 vs. Me = 18; *p* < 0.001). COVID-19 CBS severity was higher among rural residents.

The level of anxiety was differentiated by gender, age and living with someone or alone. Higher severity of anxiety symptoms was observed among women (Me = 10 vs. Me = 8; *p* < 0.001). People in early adulthood were characterized by a lower level of anxiety compared to people in late adulthood (Me = 9 vs. Me = 15; *p* = 0.015). A similar difference was observed when comparing people in middle adulthood and people in late adulthood (Me = 9 vs. Me = 15; *p* = 0.009). Furthermore, people living with somebody presented higher intensity of anxiety in comparison to people living alone (Me = 10 vs. Me = 8; *p* = 0.030).

The severity of depression symptoms was only differentiated by age. People in early adulthood were characterized by a lower level of depression symptoms compared to people in late adulthood (Me = 5 vs. Me = 14; *p* = 0.004). Also, people in middle adulthood were characterized by a lower level of symptoms of depression than the elderly (Me = 6 vs. Me = 14; *p* = 0.008). When assessing these results, the small size of the late adulthood group should be taken into account. The distribution of the size of the groups is presented in Table 1 (see also Supplementary Material).

## Discussion

The study indicates that belief in false information about the COVID-19 pandemic is strongly related to the personality tendency to believe in conspiracy theories, and that an increase in general conspiracy tendencies regarding malevolent global conspiracies, extraterrestrial cover up, personal well-being, and control of information may contribute to its development. The tendency to believe in false convictions regarding COVID-19 may be detrimental to our psychological functioning as it is associated with increased symptoms of anxiety and depression.

The strong association of COVID-19 CBS with belief in general conspiracy theories is not surprising. Indeed, it seems obvious that people who are inclined to accept and develop conspiracy theories on a variety of issues will be more likely to believe in false conspiracy theories related to the pandemic. The belief in control of information, which means the belief that the flow of information is unethically controlled by various global organisations, seems to play a particular role here. It seems that the increase in the belief in control of information, and thus in the belief in false convictions regarding COVID-19, may have been supported in the period of the SARS-CoV-2 pandemic by information uncertainty related to the new pandemic situation, which had not been known before, as well as by contradictory information and judgements about the virus that were made by authorities in the media, or by the decisions of governments during subsequent waves of illnesses that were not fully understood by citizens. The uncertainty associated with the pandemic may have contributed to the belief that public institutions provide citizens with only the information they need to create human behaviour in times of pandemic. This may be indirectly confirmed by an international study carried out by IPSOS, which observed that in many countries there was a decline in public trust with regard to the handling of the pandemic by governments (38). It is also related to another factor that reinforces the belief in false convictions regarding COVID-19, that is, the belief in malevolent global conspiracies, which means the tendency to believe that some secret group is in control of global events. Researchers have reported that a significant proportion of the public may believe that the pandemic is a planned act by those with power (39), and that the information provided may be politically motivated (40). Concepts from the field of personal well-being, which centre around conspiracy beliefs about individual health and freedom, are also an important factor that may be involved in the development of belief in false claims about the COVID-19 pandemic. The pandemic and the resulting restrictions are associated with a great deal of anxiety related specifically to values such as health and freedom. Since the start of the SARS-CoV-2 pandemic, citizens have faced anxiety about their own health and that of their loved ones, partly due to insufficient knowledge of the virus and the consequences of infection, as well as media reports of COVID-19-related infections, deaths and health complications. On the other hand, the pandemic restrictions introduced in most parts of the world have been perceived by some people as an attack on individual freedom at many levels - including individual, but also economic. The necessity of social isolation and submission to strict rules of functioning in basic areas of human activity - work, shopping, or family celebrations - was conducive to the development of frustration among citizens. Citizens were able to channel this tension in the form of criticism of the decisions of local authorities, as well as in the development of conspiracy theories about the COVID-19 pandemic - especially in the area of false information about the health consequences of the virus infection and in the area of political reasons for limiting citizens' rights.

The period of the SARS-CoV-2 virus attack is a time of widespread crisis at various levels of human functioning, from individual psychological functioning to social behaviour and mood. The experience of a pandemic, as indicated by numerous studies, is a source of anxiety tension and mental health deterioration for many people (1–6). Increased anxiety is particularly threatening as it can exacerbate a wide range of adverse phenomena during the pandemic period, such as those associated with aggression and alcohol consumption (41). The tendency to believe in false claims about the COVID-19 pandemic may be involved in the development of anxiety and depression symptoms. This means that while the tendency to believe in conspiracy theories may be an attempt to find an explanation for anxiety-producing events and through this develop an illusory understanding of them, it may also be a factor in increasing anxiety and depression symptoms among people. False beliefs about the pandemic may also motivate anti-health behaviour, making it difficult to stop the spread of the virus and treat its consequences. The danger of spreading false beliefs about COVID-19 has been recognised by the WHO, which has decided to counter these trends by publishing a list of the most popular harmful myths about the pandemic (22).

It appears that the development of belief in false claims about the COVID-19 pandemic may be a serious health risk, both in terms of preventing the spread of SARS-CoV-2 virus, as well as negative psychological consequences in the form of increased symptoms of anxiety and depression. Indeed, the assumption of false beliefs about the pandemic is associated with non-compliance with sanitary recommendations and sceptical attitudes towards vaccination (42), thus behaviours that favour the spread of the virus, more potential morbidity and death. Previous research suggests that a tendency towards conspiracy thinking is associated with low agreeableness and high openness to new experiences (43, 44). What is more, conspiracy beliefs connected with coronavirus are negatively related to proceeding in accordance with safety guidelines (45). Individuals achieving lower life satisfaction have been shown to be more likely to make false assumptions about pandemics (46). Research reports also support the positive correlation observed in our study between the severity of depressive symptoms (29) and anxiety (30) with belief in conspiracy theories about the COVID-19 pandemic in different populations.

Further investigation of the associations between belief in conspiracy theories and false convictions regarding COVID-19 vs. the severity of anxiety and depressive symptoms seems warranted to better characterise and understand the psychological functioning of a group of patients at risk of developing psychological disorders and in need of help during the COVID-19 pandemic (46).

The current findings associating belief in false convictions about the COVID-19 pandemic with increased symptoms of anxiety and depression also demonstrate the importance of providing accurate information about the pandemic and avoiding 'fake news' by opinion leaders, including but not limited to politicians, celebrities, authority figures and the media. Indeed, countering the development of belief in false claims about COVID-19 can be important for the mental health of the population.

### Limitations

The presented study, like most studies conducted in an online format, has limitations. The research was conducted using convenience sampling via social media, which allowed us to collect research material only from people who use computers and the Internet. In addition, there is a disproportion between the number of men and women in the study, resulting in women being overrepresented. The study also overwhelmingly involves people in early adulthood, while the representation of people in late adulthood is small. The results obtained can therefore mainly be related to young women with a high level of education. It should also be remembered that correlational analysis alone cannot provide definitive evidence of causality.

## Conclusions

1. The Tendency to Believe in False Information About the COVID-19 Pandemic Is Positively Related to the Tendency to Believe in Conspiracy Theories in General, and to Its Components Such as Malevolent Global Conspiracies, Extraterrestrial Cover up, Personal Well-being, Control of Information, and Government Malfeasance.

2. The Tendency to Believe in False Information Regarding the COVID-19 Pandemic may Contribute to the Development of Symptoms of Anxiety and Depression.

3. Individuals and Institutions Influencing Public Opinion Should Take Into Account That Creating Belief in False Information About the COVID-19 Pandemic can be Harmful to the Mental Health of Citizens.

4. The COVID-19 Conspiratorial Beliefs Scale, Designed to Measure the Intensity of the Tendency to Believe in False Claims About the COVID-19 Pandemic, Although in Need of Further Study, Is Initially Promising and can Help in Identifying Individuals who may Have Developed Symptoms of Anxiety and Depression due to Conspiracy Theories.

## Data Availability Statement

The original contributions presented in the study are included in the article/Supplementary Material, further inquiries can be directed to the corresponding author.

## Ethics Statement

The studies involving human participants were reviewed and approved by Bioethics Commission of the Medical University of Silesia in Katowice. Written informed consent for participation was not required for this study in accordance with the national legislation and the institutional requirements.

## Author Contributions

PD, AB, NK, MD-J, MP, and PG contributed to conception, design of the study, and wrote sections of the manuscript. PD, AB, and NK organized the database and wrote the first draft of the manuscript. PD and MD-J performed the statistical analysis. All authors contributed to manuscript revision, read, and approved the submitted version.

## Conflict of Interest

The authors declare that the research was conducted in the absence of any commercial or financial relationships that could be construed as a potential conflict of interest.

## Publisher's Note

All claims expressed in this article are solely those of the authors and do not necessarily represent those of their affiliated organizations, or those of the publisher, the editors and the reviewers. Any product that may be evaluated in this article, or claim that may be made by its manufacturer, is not guaranteed or endorsed by the publisher.

## Supplementary Material

Supplementary Table S1 Characteristics of regression models and intergroup differences.

Supplementary Data Sheet S1 Initial analysis of the psychometric properties of COVID-19 Conspiratorial Beliefs Scale.

Supplementary Data Sheet S2 COVID-19 Conspiratorial Beliefs Scale - English version.

Supplementary Data Sheet S3 COVID-19 Conspiratorial Beliefs Scale - Polish version.
