# Supplementary material for: Conspiratorial Beliefs About COVID-19 Pandemic - Can They Pose a Mental Health Risk? The Relationship Between Conspiracy Thinking and the Symptoms of Anxiety and Depression Among Adult Poles
Source: Front Psychiatry. 2022 Jun 7;13:870128. doi: 10.3389/fpsyt.2022.870128 (PMC9209766; doi:10.3389/fpsyt.2022.870128)
Supplement: Supplementary Table S1 — Characteristics of regression models and intergroup differences. [file Table_1.DOCX]

Regression models

**Table 4.** Regression model of belief in false information about the COVID-19 pandemic in the light of the tendency to believe in general conspiracy theories (*N*=700)

| Predictors | Βeta | b | b SE | p |
| --- | --- | --- | --- | --- |
| Constant |  | 4.59 | 0.59 | <.001 |
| GCBS - MGC | 0.131 | 0.31 | 0.09 | .001 |
| GCBS - ECU | 0.055 | 0.15 | 0.08 | .061 |
| GCBS - PW | 0.463 | 1.18 | 0.11 | <.001 |
| GCBS - CoI | 0.221 | 0.59 | 0.10 | <.001 |
| R²=0.62; F(4, 695)=290.32; p<.001; SEE=5.13; f^2^=1.70 | | | | |

Note: GCBS - Generic Conspiracist Beliefs Scale MGC - Malevolent Global Conspiracies, ECU - Extraterrestrial Cover Up, PW - Personal Wellbeing, CoI - Control of Information.

SE - standard error; R² - corrected R-squared; SEE - error of estimation; f^2^ - effect size for regression.

Assumptions – Statistics (*N*=700)

1. Outliers – standardized residuals (outliers outside±3 SD) (Step 1: Case 351 Std. Residual=3.99, Case 352 Std. Residual=4.11, Case 546 Std. Residual=-3.30; Step 2: Case 45 Std. Residual=3.04, Case 543 Std. Residual=-3.36, Step 3: Case 491 Std. Residual=3.01, Case 542 Std. Residual=-3.38, Step 4: Case 541 Std. Residual=3.40).

**Table 4.** Regression model of belief in false information about the COVID-19 pandemic in the light of the tendency to believe in general conspiracy theories (*N*=692)

| Predictors | Βeta | b | b SE | p |
| --- | --- | --- | --- | --- |
| Constant |  | 4.39 | 0.56 | .003 |
| GCBS - MGC | 0.11 | 0.27 | 0.09 | .027 |
| GCBS - ECU | 0.06 | 0.17 | 0.08 | <.001 |
| GCBS - PW | 0.49 | 1.23 | 0.11 | <.001 |
| GCBS - CoI | 0.22 | 0.58 | 0.10 | <.001 |
| R²=0.65; F(4, 687)=317.42; p<.001; SEE=4.92; f^2^=1.86 | | | | |

Note: GCBS - Generic Conspiracist Beliefs Scale MGC - Malevolent Global Conspiracies,
ECU - Extraterrestrial Cover Up, PW - Personal Wellbeing, CoI - Control of Information.

SE - standard error; R² - corrected R-squared; SEE - error of estimation; f^2^ - effect size for regression.

Assumptions – Statistics (*N*=692)

1. Outliers – standardized residuals (outliers outside±3 SD) no Cases outside±3 SD.
2. Normality – unstandardized residuals skewness and kurtosis (Sk=0.07, Ku=0.16)


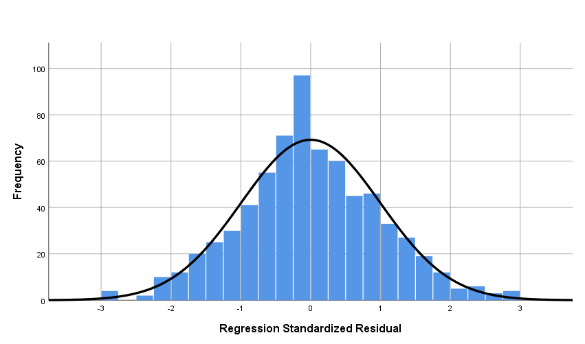


1. Homoscedasticity


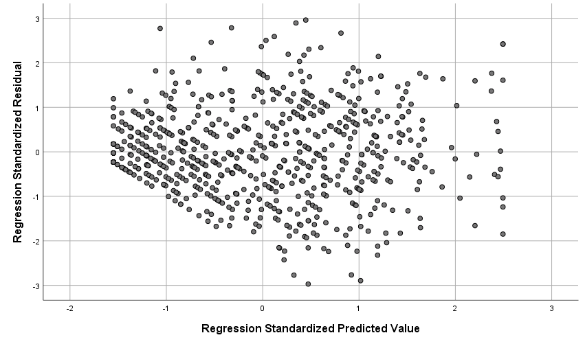


1. Independence of observations – Durbin-Watson, 2.08
2. Multicollinearity – variance inflation factor VIF (VIF_GCBS-MGC_=2.77, VIF_GCBS-ECU_=1.57, VIF _GCBS-PW_=3.52, VIF_GCBS-COI_=2.75).

**Table 5.** Regression model of anxiety and depression symptoms severity in the light of belief in false information about the COVID-19 pandemic (*N*=700)

| Predictors | Βeta | b | b SE | p |
| --- | --- | --- | --- | --- |
| HADS-A | | | | |
| Constant |  | 8.67 | 0.44 | <.001 |
| COVID-19 CBS | 0.09 | 0.04 | 0.02 | .021 |
| R²=0.01; F(1, 698)=5.32; p=.021; SEE=4.25; f^2^=0.01 | | | | |
| HADS-D | | | | |
| Constant |  | 4.72 | 0.39 | <.001 |
| COVID-19 CBS | 0.11 | 0.05 | 0.02 | .004 |
| R²=0.01; F(1, 698)=8.16; p=.004; SEE=3.70; f^2^=0.01 | | | | |

Note: COVID-19 CBS - COVID-19 Conspiratorial Beliefs Scale.

SE – standard error; R² - corrected R-squared; SEE – error of estimation; f^2^ - effect size for regression.

Assumptions – Statistics (*N*=700)

HADS-A

1. Outliers – standardized residuals (outliers outside±3 SD) no Cases outside±3 SD.
2. Normality – unstandardized residuals skewness and kurtosis (Sk=0.26, Ku=-0.37).


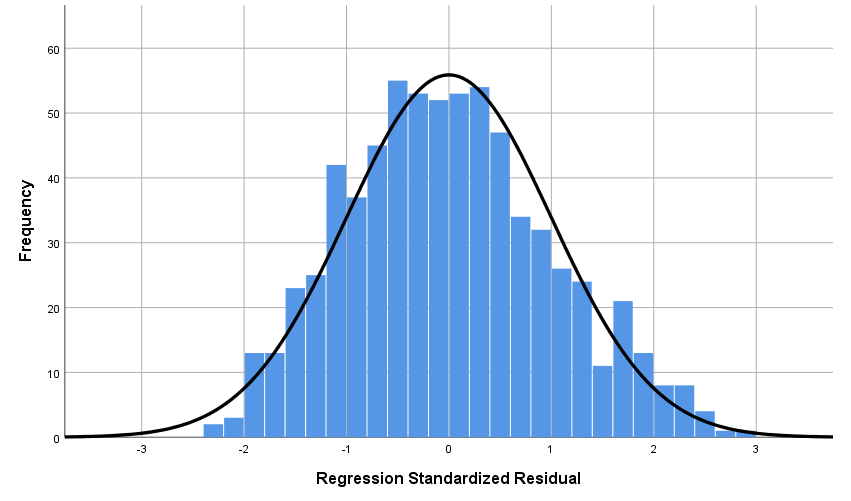


1. Homoscedasticity


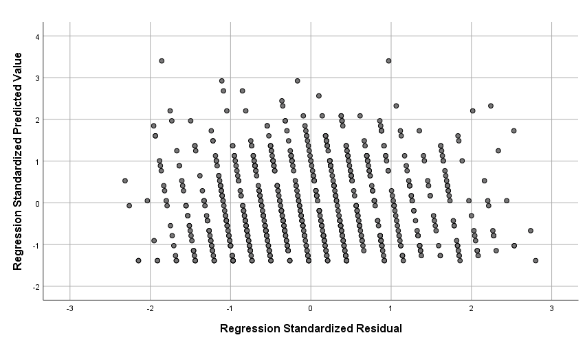


1. Independence of observations – Durbin-Watson = 1.84
2. Multicollinearity – simple regression does not assess covariance of independent variables.

HADS-D

1. Outliers – standardized residuals (outliers outside±3 SD) (Step 1: Case 48 Std. Residual=3.04, Case 49 Std. Residual=3.12, Case 115 Std. Residual=4.07; Case 354 Std. Residual=3.73, Case 501 Std. Residual=3.15, Case 659 Std. Residual=3.38, Step 2: Case 56 Std. Residual=3.10, Case 85 Std. Residual=3.06, Step 3: Case 111 Std. Residual=3.19, Case 217 Std. Residual=3.21, Case 329 Std. Residual=3.17).

**Table 5.** Regression model of anxiety and depression symptoms severity in the light of belief in false information about the COVID-19 pandemic (*N*=700 i *N*=689)

| Predictors | Βeta | b | b SE | p |
| --- | --- | --- | --- | --- |
| HADS-A | | | | |
| Constant |  | 8.67 | 0.44 | <.001 |
| COVID-19 CBS | 0.09 | 0.04 | 0.02 | .021 |
| R²=0.01; F(1, 698)=5.32; p=.021; SEE=4.25; f^2^=0.01 | | | | |
| HADS-D | | | | |
| Constant |  | 4.33 | 0.36 | <.001 |
| COVID-19 CBS | 0.14 | 0.06 | 0.02 | <.001 |
| R²=0.02; F(1, 687)=13.54; p<.001; SEE=3.42; f^2^=0.02 | | | | |

Note: COVID-19 CBS - COVID-19 Conspiratorial Beliefs Scale.

SE – standard error; R² - corrected R-squared; SEE – error of estimation; f^2^ - effect size for regression.

Assumptions – Statistics (*N*=689)

HADS-D

1. Outliers – standardized residuals (outliers outside±3 SD) no Cases outside±3 SD.
2. Normality – unstandardized residuals skewness and kurtosis (Sk=0.54, Ku=-0.11).


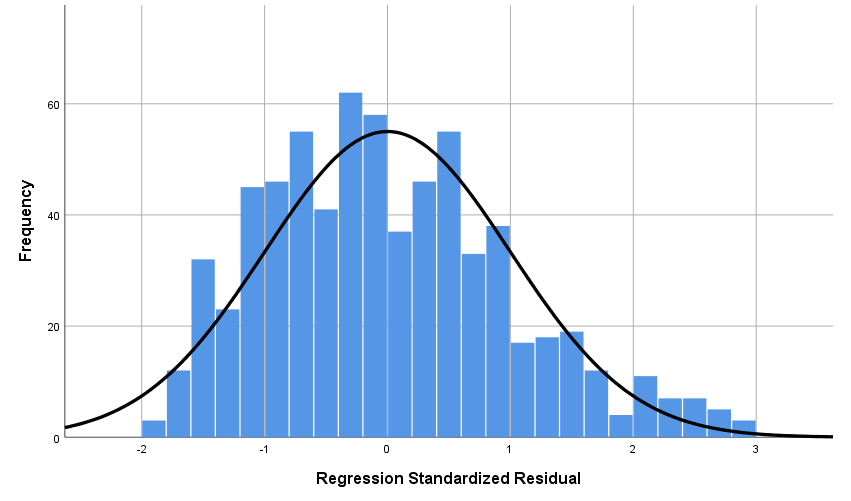


1. Homoscedasticity


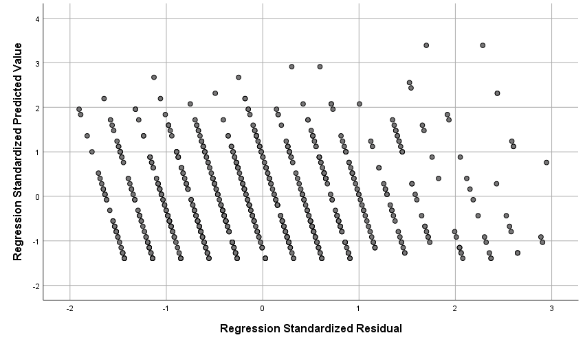


1. Independence of observations – Durbin-Watson = 2.03
2. Multicollinearity – simple regression does not assess covariance of independent variables.

Intergroup differences

COVID-19 CBS

|  |  |  | n | Me | U/H | p | 1-2 | 1-3 | 2-3 |
| --- | --- | --- | --- | --- | --- | --- | --- | --- | --- |
| Sex | 1 | Female | 585 | 20 | -0.10 | .921 |  |  |  |
|  | 2 | Male | 110 | 20 |  |  |  |  |  |
| Age | 1 | 18-29 years | 618 | 20 | 0.12 | .941 |  |  |  |
|  | 2 | 30-50 years | 75 | 20 |  |  |  |  |  |
|  | 3 | >50 years | 7 | 16 |  |  |  |  |  |
| Residence | 1 | Village | 161 | 24 | 26.96 | <.001 | .008 | <.001 | .246 |
|  | 2 | City ≤100 thous. of residents | 171 | 20 |  |  |  |  |  |
|  | 3 | City >100 thous. of residents | 368 | 18 |  |  |  |  |  |
| Education | 1 | Primary | 14 | 17 | 0.89 | .827 |  |  |  |
|  | 2 | Vocational | 4 | 19 |  |  |  |  |  |
|  | 3 | Secondary | 353 | 20 |  |  |  |  |  |
|  | 4 | Higher | 329 | 21 |  |  |  |  |  |
| Marital status | 1 | Single | 317 | 19 | 4.40 | .111 |  |  |  |
|  | 2 | In a partnership | 310 | 22 |  |  |  |  |  |
|  | 3 | Married | 73 | 20 |  |  |  |  |  |
| Living | 1 | alone | 66 | 18.5 | -1.48 | .140 |  |  |  |
|  | 2 | with somebody | 634 | 21 |  |  |  |  |  |

HADS-A

|  |  |  | n | Me | U/H | p | 1-2 | 1-3 | 2-3 |
| --- | --- | --- | --- | --- | --- | --- | --- | --- | --- |
| Sex | 1 | Female | 585 | 10 | -4.48 | <.001 |  |  |  |
|  | 2 | Male | 110 | 8 |  |  |  |  |  |
| Age | 1 | 18-29 years | 618 | 9 | 8.91 | .012 | 1.000 | .015 | .009 |
|  | 2 | 30-50 years | 75 | 9 |  |  |  |  |  |
|  | 3 | >50 years | 7 | 15 |  |  |  |  |  |
| Residence | 1 | Village | 161 | 10 | 3.72 | .156 |  |  |  |
|  | 2 | City ≤100 thous. of residents | 171 | 10 |  |  |  |  |  |
|  | 3 | City >100 thous. of residents | 368 | 9 |  |  |  |  |  |
| Education | 1 | Primary | 14 | 10 | 2.70 | .439 |  |  |  |
|  | 2 | Vocational | 4 | 9 |  |  |  |  |  |
|  | 3 | Secondary | 353 | 10 |  |  |  |  |  |
|  | 4 | Higher | 329 | 9 |  |  |  |  |  |
| Marital status | 1 | Single | 317 | 10 | 4.42 | .110 |  |  |  |
|  | 2 | In a partnership | 310 | 9 |  |  |  |  |  |
|  | 3 | Married | 73 | 10 |  |  |  |  |  |
| Living | 1 | alone | 66 | 8 | -2.17 | .030 |  |  |  |
|  | 2 | with somebody | 634 | 10 |  |  |  |  |  |

HADS-D

|  |  |  | n | Me | U/H | p | 1-2 | 1-3 | 2-3 |
| --- | --- | --- | --- | --- | --- | --- | --- | --- | --- |
| Sex | 1 | Female | 585 | 5 | -0.10 | .921 |  |  |  |
|  | 2 | Male | 110 | 5 |  |  |  |  |  |
| Age | 1 | 18-29 years | 618 | 5 | 10.13 | .006 | 1.000 | .004 | .008 |
|  | 2 | 30-50 years | 75 | 6 |  |  |  |  |  |
|  | 3 | >50 years | 7 | 14 |  |  |  |  |  |
| Residence | 1 | Village | 161 | 6 | 4.63 | .099 |  |  |  |
|  | 2 | City ≤100 thous. of residents | 171 | 6 |  |  |  |  |  |
|  | 3 | City >100 thous. of residents | 368 | 5 |  |  |  |  |  |
| Education | 1 | Primary | 14 | 7 | 7.31 | .063 |  |  |  |
|  | 2 | Vocational | 4 | 10 |  |  |  |  |  |
|  | 3 | Secondary | 353 | 6 |  |  |  |  |  |
|  | 4 | Higher | 329 | 5 |  |  |  |  |  |
| Marital status | 1 | Single | 317 | 6 | 5.36 | .069 |  |  |  |
|  | 2 | In a partnership | 310 | 5 |  |  |  |  |  |
|  | 3 | Married | 73 | 6 |  |  |  |  |  |
| Living | 1 | alone | 66 | 5 | -0.47 | .638 |  |  |  |
|  | 2 | with somebody | 634 | 5 |  |  |  |  |  |

Note: Me – median, U/H – U Mann-Whitney test, Kruskal-Wallis test statistics
