# Supplementary material for: Conspiratorial Beliefs About COVID-19 Pandemic - Can They Pose a Mental Health Risk? The Relationship Between Conspiracy Thinking and the Symptoms of Anxiety and Depression Among Adult Poles
Source: Front Psychiatry. 2022 Jun 7;13:870128. doi: 10.3389/fpsyt.2022.870128 (PMC9209766; doi:10.3389/fpsyt.2022.870128)
Supplement: Supplementary Data Sheet S1 — Initial analysis of the psychometric properties of COVID-19 Conspiratorial Beliefs Scale. [file Data_Sheet_1.PDF]

Initial analysis of the psychometric properties of

COVID-19 Conspirational Beliefs Scale

(tested on a sample of 700 people)

Paweł Dębski[1*], Adrianna Boroń[1], Natalia Kapuśniak[1], Małgorzata Dębska-Janus[2], Magdalena Piegza[1]

1. Department of Psychiatry, Faculty of Medical Sciences in Zabrze, Medical University of Silesia in Katowice, Pyskowicka 49, 42-612 Tarnowskie Góry, Poland; psychiatriatarnowskiegory@sum.edu.pl

2. Institute of Sport Sciences, The Jerzy Kukuczka Academy of Physical Education in Katowice, Mikołowska 72A, 40-065 Katowice, Poland; m.debska@awf.katowice.pl

* Correspondence: Paweł Dębski, PhD, Department of Psychiatry, Faculty of Medical Sciences in Zabrze, Medical University of Silesia in Katowice, Pyskowicka 49, 42-612 Tarnowskie Góry, Poland, pdebski@sum.edu.pl

COVID-19 CBS ITEMS

No.	linguistic content
**1**	Vitamins and minerals supplementation can cure SARS-CoV-2 infection
**2**	Wearing face masks causes oxygen deficiency and carbon dioxide poisoning
**3**	Consumption of alcohol protects from COVID-19 infection
**4**	The spread of the 5G mobile network is related to the spread of SARS-CoV-2 virus
**5**	There is a drug that can effectively cure COVID-19 patients, but information about it is confidential and inaccessible to ordinary people
**6**	SARS-CoV-2 was created by a man using genetic engineering techniques, to serve as a biological weapon
**7**	Governments deliberately spread false information about COVID-19 in order to conceal the actual state of the pandemic
**8**	The SARS-CoV-2 pandemic does not exist and it was invented by a group of people benefiting from it
**9**	Health workers receive financial benefits for diagnosing COVID-19 or listing COVID-19 as the cause of death
**10**	SARS-CoV-2 tests are unreliable, they may be positive in the case of infection with another virus

TEST ITEM ANALYSIS

(the significance of differences between extreme groups)

A study of the significance of differences between extreme groups was used, creating groups containing 27% of results from the ends of the distribution of results (N=700). Student's t-test was used.

Item	27% of low scores		27% of high scores		t	p
	Mean 1	SD 1	Mean 2	SD 2		
**1**	1,000	0,000	3,574	0,676	-52,310	0,00
**2**	1,312	0,465	4,848	0,360	-82,997	0,00
**3**	1,000	0,000	4,555	0,498	-98,083	0,00
**4**	1,000	0,000	3,267	1,045	-29,831	0,00
**5**	1,238	0,427	4,665	0,473	-74,080	0,00
**6**	1,000	0,000	4,199	0,651	-67,589	0,00
**7**	1,000	0,000	3,871	0,803	-48,209	0,00
**8**	1,000	0,000	2,246	1,070	-16,013	0,00
**9**	1,000	0,000	2,215	1,170	-14,269	0,00
**10**	1,000	0,000	3,555	0,730	-48,135	0,00

The test items significantly differentiate the extreme groups.

EXPLORATORY FACTOR ANALYSIS

Kaiser criterion in factor analysis.

Values	Eigenvalues: main components			
	Eigenvalues	% Variance	Cumulative eigenvalue	Cumulative %
**1**	**4,784**	**47,840**	**4,784**	**47,840**
**2**	**1,247**	**12,468**	**6,031**	**60,308**
**3**	0,751	7,511	6,782	67,819
**4**	0,611	6,114	7,393	73,933
**5**	0,558	5,575	7,951	79,508
**6**	0,471	4,712	8,422	84,220
**7**	0,429	4,286	8,851	88,506

**8**	0,410	4,105	9,261	92,610
**9**	0,388	3,884	9,649	96,494
**10**	0,351	3,506	10,000	100,000

The Kaiser criterion revealed that the two factors > 1, indicating the two-factor nature of the test.

Cattel scree plot test.

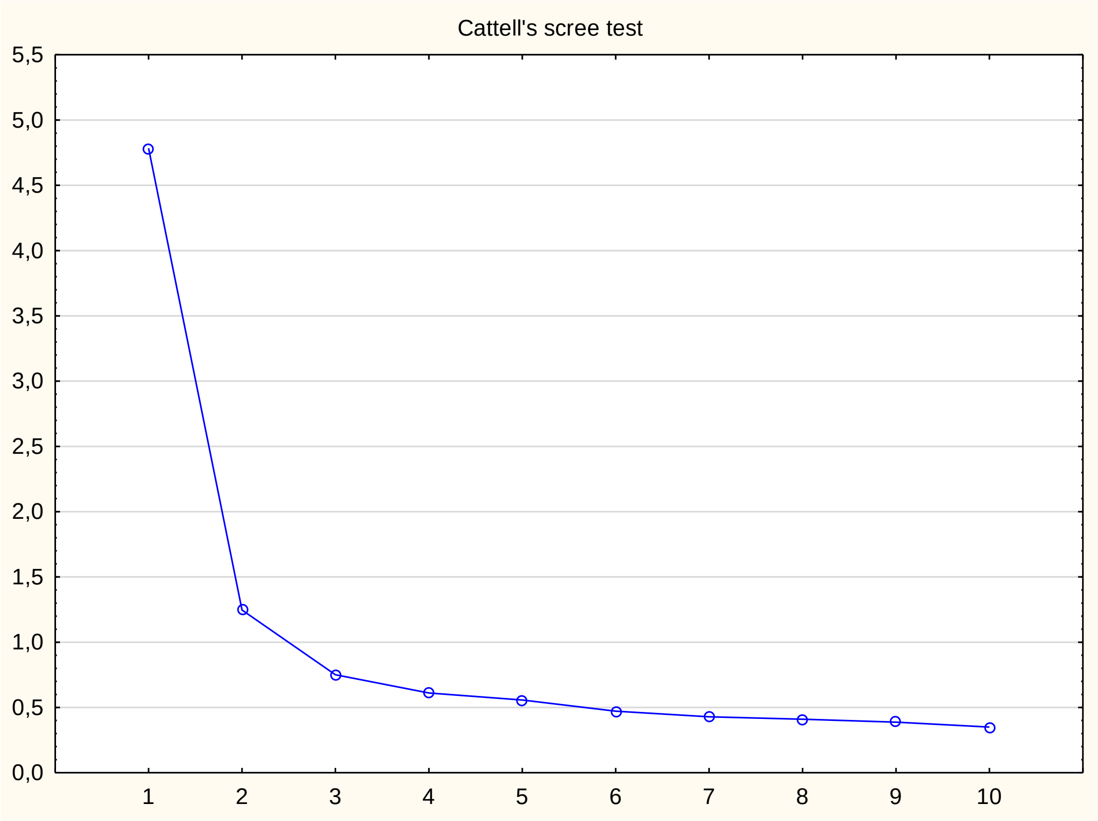

Cattel's scree plot test suggests a two-factor nature of the test.

Factor loadings assuming a two-factor structure obtained by the Varimax method.

Variable	Factor loadings assuming two factors (Varimax sur)	
	**Factor 1**	**Factor 2**
**1**	0,275	**0,570**
**2**	0,463	**0,625**
**3**	0,032	**0,788**
**4**	0,197	**0,792**
**5**	**0,641**	0,422
**6**	**0,724**	0,325

**7**	**0,808**	0,108
**8**	0,461	**0,584**
**9**	**0,764**	0,183
**10**	**0,816**	0,121
**Explained variance**	3,381	2,649
**Participation**	0,338	0,265

On the basis of factor analysis, assuming a two-factor test, it was decided to include items 5, 6, 7, 9, 10 to the first factor, which was called: conspiracy beliefs about the harmful effects of humans in the COVID-19 pandemic, while items 1, 2, 3, 4, 8 were included in the second factor, which was called: false beliefs about prevention and treatment in the conditions of the COVID-19 pandemic.

RELIABILITY

The reliability of the entire test was estimated using the Cronbach's α coefficient.

Variable	Mean without	Variance without	SD without	item-test correlation	α coefficient without
**1**	19,487	59,996	7,746	0,475	0,871
**2**	19,707	56,844	7,54	0,663	0,858
**3**	20,247	63,675	7,98	0,416	0,874
**4**	20,239	62,356	7,897	0,546	0,868
**5**	19,534	55,255	7,433	0,679	0,856
**6**	19,119	54,433	7,378	0,692	0,855
**7**	18,544	54,982	7,415	0,622	0,861
**8**	19,903	57,565	7,587	0,63	0,86
**9**	18,963	53,447	7,311	0,636	0,86
**10**	18,464	54,337	7,371	0,642	0,859

Scale Summary: mean = 21.579; SD = 8.358; Cronbach's α: 0.875; standardized alpha: 0.764; average correlation between items: 0.421

The Cronbach's α coefficient for the entire test was 0.875.

Factor 1 Reliability - conspiracy beliefs about the harmful effects of humans in the COVID-19 pandemic.

Variable	Mean without	Variance without	SD without	item-test correlation	α coefficient without
**5**	11,224	21,083	4,592	0,634	0,828
**6**	10,809	20,155	4,489	0,689	0,814
**7**	10,234	19,831	4,453	0,674	0,818
**9**	10,653	19,309	4,394	0,645	0,827
**10**	10,154	19,633	4,431	0,677	0,817

Scale Summary: mean = 13.269; SD = 5.493; Cronbach's α: 0.851; standardized alpha: 0.853; average correlation between items: 0.538

The Cronbach's α coefficient for the factor 1 was 0.851.

Factor 2 Reliability - false beliefs about prevention and treatment in the conditions of the COVID-19 pandemic.

Variable	Mean without	Variance without	SD without	item-test correlation	α coefficient without
**1**	6,219	9,217	3,036	0,492	0,766
**2**	6,439	8,369	2,893	0,634	0,713
**3**	6,979	10,612	3,258	0,505	0,759
**4**	6,970	10,215	3,196	0,621	0,730
**8**	6,634	8,695	2,949	0,590	0,729

Scale Summary: mean = 8.310; SD = 3.739; Cronbach's α: 0.781; standardized alpha: 0.791; average correlation between items: 0.434

The Cronbach's α coefficient for the factor 2 was 0.781.
