# Supplementary material for: Conspiratorial Beliefs About COVID-19 Pandemic - Can They Pose a Mental Health Risk? The Relationship Between Conspiracy Thinking and the Symptoms of Anxiety and Depression Among Adult Poles
Source: Front Psychiatry. 2022 Jun 7;13:870128. doi: 10.3389/fpsyt.2022.870128 (PMC9209766; doi:10.3389/fpsyt.2022.870128)
Supplement: Supplementary Data Sheet S2 — COVID-19 Conspiratorial Beliefs Scale - English version. [file Data_Sheet_2.PDF]

COVID-19 Conspirational Beliefs Scale

Paweł Dębski, Adrianna Boroń, Natalia Kapuśniak, Małgorzata Dębska, Magdalena Piegza

Instruction

Use the options below to determine your opinion on the following statements

1. *I strongly disagree*
2. *I tend to disagree*
3. *I cannot decide if it is true or not*
4. *I tend to agree*
5. *I strongly agree*

No.	**COVID-19 CBS**<br>Paweł Dębski, Adrianna Boroń, Natalia Kapuśniak, Małgorzata Dębska, Magdalena Piegza	Your opinion
1	Vitamins and minerals supplementation can cure SARS-CoV-2 infection	1 – 2 – 3 – 4 – 5
2	Wearing face masks causes oxygen deficiency and carbon dioxide poisoning	1 – 2 – 3 – 4 – 5
3	Consumption of alcohol protects from COVID-19 infection	1 – 2 – 3 – 4 – 5
4	The spread of the 5G mobile network is related to the spread of SARS-CoV-2 virus	1 – 2 – 3 – 4 – 5
5	There is a drug that can effectively cure COVID-19 patients, but information about it is confidential and inaccessible to ordinary people	1 – 2 – 3 – 4 – 5
6	SARS-CoV-2 was created by a man using genetic engineering techniques, to serve as a biological weapon	1 – 2 – 3 – 4 – 5

7	Governments deliberately spread false information about COVID-19 in order to conceal the actual state of the pandemic	1 – 2 – 3 – 4 – 5
8	The SARS-CoV-2 pandemic does not exist and it was invented by a group of people benefiting from it	1 – 2 – 3 – 4 – 5
9	Health workers receive financial benefits for diagnosing COVID-19 or listing COVID-19 as the cause of death	1 – 2 – 3 – 4 – 5
10	SARS-CoV-2 tests are unreliable, they may be positive in the case of infection with another virus	1 – 2 – 3 – 4 – 5
