# Supplementary material for: Conspiratorial Beliefs About COVID-19 Pandemic - Can They Pose a Mental Health Risk? The Relationship Between Conspiracy Thinking and the Symptoms of Anxiety and Depression Among Adult Poles
Source: Front Psychiatry. 2022 Jun 7;13:870128. doi: 10.3389/fpsyt.2022.870128 (PMC9209766; doi:10.3389/fpsyt.2022.870128)
Supplement: Supplementary Data Sheet S3 — COVID-19 Conspiratorial Beliefs Scale - Polish version. [file Data_Sheet_3.PDF]

COVID-19 CBS Skala Fałszywych Przekonań

(COVID-19 Conspirational Beliefs Scale)

Paweł Dębski, Adrianna Boroń, Natalia Kapuśniak, Małgorzata Dębska, Magdalena Piegza

Instrukcja (*Instruction*)

Określ swoje zdanie na temat poniższych stwierdzeń posługując się poniższymi możliwościami:

(*Use the options below to determine your opinion on the following statements*)

1. Zdecydowanie się nie zgadzam (*I strongly disagree*)
2. Raczej się nie zgadzam (*I tend to disagree*)
3. Nie potrafię zdecydować czy to prawda czy nie (*I cannot decide if it is true or not*)
4. Raczej się zgadzam (*I tend to agree*)
5. Zdecydowanie się zgadzam (*I strongly agree*)

No.	**COVID-19 CBS**<br>Paweł Dębski, Adrianna Boroń, Natalia Kapuśniak, Małgorzata Dębska, Magdalena Piegza	Twoje zdanie<br>(*Your opinion*)
1	Suplementacja witamin i substancji mineralnych może wyleczyć zakażenie SARS-CoV-2<br>(*Vitamins and minerals supplementation can cure SARS-CoV-2 infection*)	1 – 2 – 3 – 4 – 5
2	Prawidłowe użycie masek powoduje niedobór tlenu oraz zatrucie dwutlenkiem węgla<br>(*Wearing face masks causes oxygen deficiency and carbon dioxide poisoning*)	1 – 2 – 3 – 4 – 5
3	Spożywanie alkoholu chroni przed zakażeniem COVID-19<br>(*Consumption of alcohol protects from COVID-19 infection*)	1 – 2 – 3 – 4 – 5
4	Rozpowszechnienie sieci mobilnej 5G ma związek z roznoszeniem się wirusa SARS-CoV-2<br>(*The spread of the 5G mobile network is related to the spread of SARS-CoV-2 virus*)	1 – 2 – 3 – 4 – 5
5	Istnieje lekarstwo, które skutecznie może wyleczyć chorych na COVID-19, ale informacje o nim są utajnione i jest ono niedostępne dla zwykłych ludzi	1 – 2 – 3 – 4 – 5

	(*There is a drug that can effectively cure COVID-19 patients, but information about it is confidential and inaccessible to ordinary people*)	
6	SARS-CoV-2 został stworzony przez człowieka za pomocą technik inżynierii genetycznej, aby służyć jako broń biologiczna (*SARS-CoV-2 was created by a man using genetic engineering techniques, to serve as a biological weapon*)	1 – 2 – 3 – 4 – 5
7	Rządy państw celowo rozpowszechniają fałszywe informacje na temat COVID-19, aby zataić faktyczny stan pandemii (*Governments deliberately spread false information about COVID-19 in order to conceal the actual state of the pandemic*)	1 – 2 – 3 – 4 – 5
8	Pandemia SARS-CoV-2 nie istnieje, została wymyślona przez grupę ludzi, która czerpie z niej korzyści (*The SARS-CoV-2 pandemic does not exist and it was invented by a group of people benefiting from it*)	1 – 2 – 3 – 4 – 5
9	Pracownicy ochrony zdrowia otrzymują korzyści finansowe za zdiagnozowanie COVID-19/wpisanie jako przyczynę zgonu COVID-19 (*Health workers receive financial benefits for diagnosing COVID-19 or listing COVID-19 as the cause of death*)	1 – 2 – 3 – 4 – 5
10	Testy w kierunku SARS-CoV-2 są niemiarodajne, mogą być pozytywne w przypadku zakażenia innym wirusem (*SARS-CoV-2 tests are unreliable, they may be positive in the case of infection with another virus*)	1 – 2 – 3 – 4 – 5
